# Transcranial Alternating Current Stimulation (tACS) Enhances Mental Rotation Performance during and after Stimulation

**DOI:** 10.3389/fnhum.2017.00002

**Published:** 2017-01-31

**Authors:** Florian H. Kasten, Christoph S. Herrmann

**Affiliations:** ^1^Experimental Psychology Lab, Department of Psychology, European Medical School, Cluster for Excellence “Hearing for all”, Carl von Ossietzky UniversityOldenburg, Germany; ^2^Research Center Neurosensory Science, Carl von Ossietzky UniversityOldenburg, Germany

**Keywords:** transcranial alternating current stimulation (tACS), transcranial electrical stimulation (TES), aftereffect, EEG, alpha oscillations, event-related-desynchronization (ERD), mental rotation

## Abstract

Transcranial alternating current stimulation (tACS) has been repeatedly demonstrated to modulate endogenous brain oscillations in a frequency specific manner. Thus, it is a promising tool to uncover causal relationships between brain oscillations and behavior or perception. While tACS has been shown to elicit a physiological aftereffect for up to 70 min, it remains unclear whether the effect can still be elicited if subjects perform a complex task interacting with the stimulated frequency band. In addition, it has not yet been investigated whether the aftereffect is behaviorally relevant. In the current experiment, participants performed a Shepard-like mental rotation task for 80 min. After 10 min of baseline measurement, participants received either 20 min of tACS at their individual alpha frequency (IAF) or sham stimulation (30 s tACS in the beginning of the stimulation period). Afterwards another 50 min of post-stimulation EEG were recorded. Task performance and EEG were acquired during the whole experiment. While there were no effects of tACS on reaction times or event-related-potentials (ERPs), results revealed an increase in mental rotation performance in the *stimulation* group as compared to sham both during and after stimulation. This was accompanied by increased ongoing alpha power and coherence as well as event-related-desynchronization (ERD) in the alpha band in the *stimulation* group. The current study demonstrates a behavioral and physiological aftereffect of tACS in parallel. This indicates that it is possible to elicit aftereffects of tACS during tasks interacting with the alpha band. Therefore, the tACS aftereffect is suitable to achieve an experimental manipulation.

## Introduction

Transcranial alternating current stimulation (tACS) is a relatively novel method that has been demonstrated to modulate endogenous brain oscillations in a frequency specific manner (Herrmann et al., 2013; Reato et al., 2013; Helfrich et al., 2014b). By applying weak sinusoidal currents on the scalp, tACS is thought to entrain spontaneous brain oscillations in the range of the stimulation frequency, rendering it a promising tool to investigate causal relationships between these oscillations and cognitive functions (Thut et al., 2011; Fröhlich, 2015; Antal and Herrmann, 2016; Herrmann et al., 2016;). Numerous studies investigated effects of tACS on perception (Kanai et al., 2008; Laczó et al., 2012; Strüber et al., 2014), behavior (Antal et al., 2008; Sela et al., 2012; Brignani et al., 2013) and cognitive functions (Lustenberger et al., 2015; Vosskuhl et al., 2015; Chander et al., 2016). A recent meta-analysis found tACS to reliably induce enhancing effects on cognitive performance and perception with overall effect sizes in the small to moderate range (Schutter and Wischnewski, 2016). Furthermore, they found individually tailored, EEG guided stimulation frequencies (i.e., at participants’ individual alpha frequency, IAF) and anterior-posterior montages with intensities larger or equal to 1 mA to be beneficial for the size of the stimulation effect (Schutter and Wischnewski, 2016).

Directly monitoring physiological effects of tACS during application remains challenging due to the massive artifact that is introduced to the M/EEG signals. First attempts to reconstruct brain activity during tACS have been made using a variety of methods. For example, Helfrich et al. (2014b) applied a combination of template subtraction and principal component analysis (PCA). Neuling et al. (2015) reconstructed MEG signals using a linearly constrained minimum variance beamformer filter. Other researches applied alternative waveforms for stimulation such as sawtooths (Dowsett and Herrmann, 2016) or amplitude modulated sine waves (Witkowski et al., 2016). However, some of these methods have not been without criticism (Noury et al., 2016). While most behavioral studies rely upon online effects of tACS on behavioral measures, a large proportion of physiological studies conducted in humans measured outlasting effects of tACS in the EEG. This aftereffect is consistently reported for a variety of measures and frequency bands (Neuling et al., 2013; Wach et al., 2013; Helfrich et al., 2014a; Vossen et al., 2015; for an overview see Veniero et al., 2015) and has recently been demonstrated to last for up to 70 min after stimulation in the alpha band (Kasten et al., 2016). Considering this long lasting effect, it is desirable to make use of the aftereffect in experimental designs offering the opportunity to measure artifact-free M/EEG signals in parallel to task performance without the need for sophisticated procedures for artifact removal. However, up to now the aftereffect has merely been observed in isolation while subjects performed simple auditory or visual vigilance tasks causing as little interference with the stimulated brain oscillation as possible (Zaehle et al., 2010; Neuling et al., 2013; Vossen et al., 2015; Kasten et al., 2016). Thus, it remains unclear whether a similar aftereffect can still be induced (or measured) if participants are engaged in a more complex task, causing stronger modulations of the stimulated frequency bands themselves. It is known, for example, that task complexity and cognitive load modulate event-related-desynchronization (ERD) patterns in the alpha band (Van Winsun et al., 1984; Boiten et al., 1992; Dujardin et al., 1995). Furthermore, it is largely unclear whether the elicited physiological changes affect behavioral measures such as reaction times or task performance. This is especially crucial for clinical applications of tACS where long lasting stimulation effects are required to effectively recover dysfunctional oscillations, which are implicated in several neurological and psychiatric conditions (Herrmann and Demiralp, 2005; Uhlhaas and Singer, 2006, 2012).

The current study aimed to measure both behavioral and physiological aftereffects of tACS while participants performed a mental rotation task as introduced by Shepard and Metzler (1971). Since their groundbreaking experiment, mental rotation has been excessively studied. One of the first and most robust findings was the almost linear relationship between reaction times and rotation angle which has been shown to be independent of stimulus complexity and the dimension in which the object has to be rotated (Shepard and Metzler, 1971; Cooper, 1975). Furthermore, mental rotation is one of the few domains where sex differences are consistently reported, suggesting that males tend to outperform females (Linn and Petersen, 1985; Voyer et al., 1995). Mental rotation performance is widely used as a measure of cognitive performance and has been linked to alpha and theta oscillations in human M/EEG (Doppelmayr et al., 2002; Klimesch et al., 2003; Hanslmayr et al., 2005; Johnson and Bouchard, 2005). While theta oscillations appear to synchronize during mental rotation, alpha oscillations desynchronize as compared to a reference period prior to stimulus onset (Michel et al., 1994; Klimesch et al., 2007b). A phenomenon referred to as ERD/ERS (event-related desynchronization/synchronization). Stronger ERD in the alpha band has been shown to be related to higher cognitive performance especially in visual-spatial and memory tasks (Neubauer et al., 1995; Klimesch, 1999; Doppelmayr et al., 2002). Michel et al. (1994) found the duration of ERD during mental rotation to increase with the angle objects have to be mentally rotated. Additional evidence supporting the functional role of alpha desynchronization during mental rotation arises from studies using neurofeedback training (NFT) and repetitive transcranial magnetic stimulation (rTMS). In these studies, ERD in the alpha band was increased by enhancing alpha power in a reference period before stimulus onset (Klimesch et al., 2003; Hanslmayr et al., 2005; Zoefel et al., 2011). While the NFT experiments utilized posterior electrodes to provide feedback about subjects alpha activity (Klimesch et al., 2003; Hanslmayr et al., 2005; Zoefel et al., 2011) applied rTMS over the frontal and right parietal cortex. The elicited changes on participants’ alpha power/ERD were accompanied by enhanced task performance. In contrast, reaction times were not affected in these experiments. In summary, results suggest that on the one hand mental rotation performance depends on neural oscillations in the alpha band and their desynchronization during task execution. On the other hand, the desynchronization of alpha oscillations during task execution constitute regular modulations/distortions of alpha oscillations. Those can possibly distort or shorten tACS induced aftereffects. Due to these physiological properties, mental rotation is well suited to evaluate the robustness of the tACS aftereffect (the possibility to induce aftereffects in the presence of strong interference in the stimulated frequency band). It should be noted, however, that the current study did not aim to systematically evaluate the effect of different degrees of complexity on the tACS aftereffect, but rather tested whether the effects reported during resting measurements can in principle also be induced in a more complex setting.

In order to achieve a broad characterization of the physiological and behavioral changes following tACS, the current study carried out various measures to quantify the aftereffect of tACS. Besides task performance and reaction times, we analyzed ongoing alpha power during mental rotation and resting periods as well as the mean magnitude squared coherence of ongoing alpha activity. Both measures have been used to quantify outlasting effects of tACS in the past. Several studies found increased alpha power after tACS (Zaehle et al., 2010; Neuling et al., 2013; Vossen et al., 2015; Kasten et al., 2016) during resting state measurements. We expected similar patterns in our experiment during mental rotation and during resting periods. With regard to coherence, Neuling et al. (2013) and Helfrich et al. (2014a) reported outlasting effects of tACS on interhemispheric coherence. However, Neuling et al. (2013) found this effect only during eyes-closed measurement but not during eyes-open and suggested tACS effects to depend on brain-state. In the current analysis we tested whether interhemispheric coherence is increased for a subset of EEG electrodes during mental rotation. In addition, we evaluated event-related measures namely ERD in the alpha band and event-related potentials (ERPs). By increasing ongoing alpha power we also expected ERD in the alpha band to be increased after tACS compared to sham, as there is higher alpha power to desynchronize from when a stimulus is presented. We hypothesized this increase in ERD to be accompanied by enhanced performance in the mental rotation task in the stimulation group as compared to sham, but no changes in reaction times. However, for performance during tACS we expected a different pattern. Recent experiments (Neuling et al., 2015; Vosskuhl et al., 2016) suggested decreased ERD in the alpha band in response to visual stimulation during the application of tACS. Unfortunately, this was not explicitly tested or just indirectly inferred from reduced event-related BOLD response, respectively. Thus, these findings have to be interpreted with caution. Nevertheless, based on the available, albeit sparse evidence and the principles of entrainment, it seems reasonable to hypothesize tACS to reduce or overwrite ERD by entraining oscillations before and after stimulus presentation. Thus, we supposed performance in the mental rotation task to be reduced in the stimulation group compared to sham during the application of tACS due to this reduction in ERD. The analysis of ERPs was rather exploratory. However, latency and amplitude of P1 and N1 components of ERPs have been demonstrated to be (at least in part) generated by evoked oscillations in the alpha range (Gruber et al., 2005; Klimesch et al., 2007a). Thus, the amplitude of these components might be enhanced after tACS in the alpha band.

## Materials and Methods

**Figure 1:**
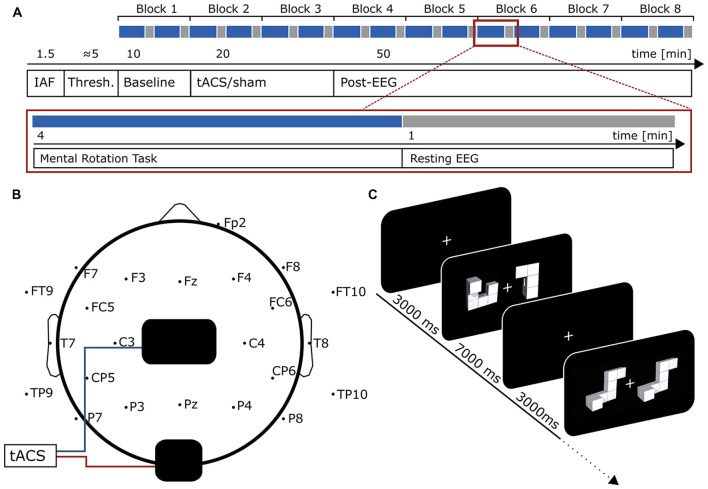
**Experimental Design. (A)** Time-course of the experiment. In the beginning, 90 s of eyes-closed EEG was recorded to determine participants individual alpha frequency (IAF). Afterwards, tACS intensity was adjusted to participants’ sensation threshold before the actual experiment started. First, 10 min of baseline measurement were acquired. During the whole experiment participants performed a mental rotation task intermitted by 1 min resting EEG every 24 trials (4 min, red box, blue indicates mental rotation period, gray resting EEG). During resting EEG, participants performed a visual vigilance task. Each block consisted of two mental rotation and two resting periods. The baseline measurement was followed by 20 min of tACS or sham stimulation and 50 min of post-stimulation EEG. **(B)** Electrode setup. tACS electrodes (black) were positioned centered above Cz and Oz. EEG was measured from 23 positions following the international 10–10 system with electrode sites above or close to tACS electrodes left blank. **(C)** Mental rotation task. Each trial started with the presentation of a white fixation cross at the center of the screen. After 3000 ms the mental rotation stimulus display (taken from Ganis and Kievit, 2015) appeared and remained on screen for another 7000 ms. During this time, participants were asked to judge whether the two presented figures were identical (but rotated) or different. The first display contains an example for a target differing from the cue, the second for a target similar to the cue.

Twenty-three healthy subjects reporting no history of neurological or psychiatric disease received either 20 min of tACS or sham stimulation during the experiment (Figure 1A). Participants were medication-free at the day of measurement and gave written informed consent prior to the experiment. They were especially informed about the applied methods (EEG/tACS) and potential risks of the electrical stimulation. After signing the consent form participants filled out a questionaire assessing exclusion criteria for the experiment (especially psychiatric and neurological conditions and metal items/implants inside or outside the head). All were right-handed according to the Edinburg handedness-scale (Oldfield, 1971). Subjects were randomly assigned to either stimulation or sham group. Both groups were counterbalanced for participants’ sex and time of measurement (sessions started at 10 AM or 2 PM). Data from six subjects had to be discarded. Two datasets were corrupted due to technical difficulties, three participants did not comply with the instructions or exhibited chance-level performance in the mental rotation task. A recent study suggested tACS to be only effective with low baseline power in the to-be stimulated frequency band (Neuling et al., 2013). To avoid non-responsiveness to the stimulation due to such ceiling effects, power in the IAF ± 2 Hz band during the baseline measurement was *z*-transformed. One subject exhibited a *z*-score above 1.65 (corresponding to an α-level <0.05, one-tailed) and was excluded from further analysis. Thus, 17 participants (8 females, age: 23.41 ± 3.28 years) remained for analysis (9 in sham, 8 in stimulation group). The experiment was approved by the local ethics committee at the University of Oldenburg and conducted in accordance with the declaration of Helsinki.

### EEG

Participants were seated in a recliner in an electrically shielded, dark room. EEG was acquired from 24 active Ag-AgCl Electrodes (ActiCap, Brain Products, Gilching, Germany), following the international 10–10 system. Electrode positions close to stimulation electrodes were left blank (Figure 1B). Ground electrode was placed at AFz. Position Fp1 served as reference which is the standard configuration of the ActiCap system. In addition, a vertical EOG was recorded from an electrode below the right eye. All impedances were kept below 20 kΩ. Data were digitized at a rate of 10 kHz using a 24-bit ActiChamp amplifier and stored on a computer using BrainVision PyCorder software (both Brain Products, Gilching, Germany).

Prior to the main experiment, 90 s of eyes-closed resting EEG were recorded to determine participants’ IAF, which was later used as stimulation frequency. EEG was split into 1 sec segments and fast Fourier transformations (FFTs) were computed on the epochs. The resulting frequency-spectra were averaged and the power peak in the 8–12 Hz range at electrode Pz was used as stimulation frequency. If no peak was evident the measurement was repeated. After stimulation intensity was adjusted to participants’ sensation threshold (see “Electrical Stimulation” Section), the main-experiment started. The experiment consisted of 10 min baseline measurement, 20 min tACS or sham stimulation and another 50 min of post-stimulation EEG measurement (Figure 1A). Participants performed a mental rotation task similar to the classic experiment of Shepard and Metzler (1971) intermitted by 1 min resting periods throughout the experiment (Figures 1A,C).

### Electrical Stimulation

tACS was delivered by a battery-operated stimulator system (DC Stimulator Plus, Neuroconn, Illmenau, Germany). Two surface conductive rubber electrodes were attached to participants’ scalp using an adhesive, electrically conductive paste (ten20 conductive paste, Weaver and Co., Aurora, CO, USA). Electrodes were positioned centered above Cz (5 cm × 7 cm) and Oz (4 cm × 4 cm; Figure 1B). This montage has been shown to achieve highest current densities in posterior brain regions in modeling studies (Neuling et al., 2012b) and has successfully been used in previous studies to elicit aftereffects in the alpha band (Neuling et al., 2013; Kasten et al., 2016). Furthermore, previous experiments using TMS or NFT targeted similar brain areas to enhance mental rotation performance (Klimesch et al., 2003; Hanslmayr et al., 2005; Zoefel et al., 2011). The sinusoidal current was digitally generated using Matlab 2012b (The MathWorks Inc., Natick, MA, USA), send to a digital-analog converter (Ni USB 6229, National Instruments, Austin, TX, USA) and streamed to the remote input of the stimulator. Electrode impedance was kept below 10 kΩ. Participants were stimulated at their IAF (9.82 Hz ± 1.2 Hz) with intensities adjusted to their individual sensation threshold (900 μA ± 335 μA); defined as the highest intensity at which participants’ did not notice the stimulation (i.e., no phosphene or skin sensation). The thresholding was performed to rule out confounding effects of sensations such as phosphenes or tingling during stimulation. After 10 min of baseline measurement the stimulation group received 20 min of tACS with 10 s fade-in and fade-out in the beginning and the end of the stimulation. While all other parameters were kept the same, the sham group received only 30 s of tACS (including 10 s fade-in and fade-out) at the beginning of the stimulation period.

### Mental Rotation Task

Before, during and after stimulation, participants performed a mental rotation task similar to the original experiment of Shepard and Metzler (1971). The task was presented on a computer screen (Samsung SyncMaster P247GH, 1920 × 1080 pixels, 60 Hz refresh rate) at a distance of approximately 100 cm using Psychtoolbox 3 (Kleiner et al., 2007) running on Matlab 2012b (The MathWorks Inc., Natick, MA, USA). Stimuli were taken from a recently published open-source stimulus-set (Ganis and Kievit, 2015) consisting of 48 three-dimensional objects and a total of 384 stimulus displays. Each display contains a cue object on the left and a target object on the right side which is rotated by either 0, 50, 100 or 150° on the vertical axis. The target object can be either identical to the cue (but rotated) or different; i.e., mirrored or partly mirrored in addition to the rotation, such that the two figures cannot be brought in alignment by solely rotating them (see Ganis and Kievit, 2015 for detailed descriptions and example figures). Stimuli were presented randomly in eight blocks each comprised of 48 stimulus displays (800 × 427 pixels) with the constraint that each block contained equal numbers of rotation angles and displays containing identical and different objects. All trials started with the presentation of a fixation cross at the center of the screen. After 3000 ms a stimulus display was presented and remained on screen for 7000 ms (Figure 1C). Participants were asked to judge whether the target stimulus was identical or different to the cue by pressing a button with their left (identical) or right (different) index finger. They were instructed to answer as fast and accurate as possible. The time window to respond was equal to the duration of the stimulus presentation.

Every 24 trials the mental rotation task was interrupted by a 1 min resting period. To ensure participants remained attentive, a visual vigilance task similar to previous studies (Zaehle et al., 2010; Kasten et al., 2016) was implemented. A fixation cross was presented at the center of the screen and rotated by 45° for 500 ms. Stimulus onset was jittered between 30 and 40 s after beginning of the trial. Participants had to react to the rotation by pressing one of the response buttons within 2 s after stimulus onset.

The first block (48 trials) served as baseline measurement before stimulation, the two subsequent blocks (96 trials) were performed during the application of tACS or sham stimulation. The remaining five blocks (240 trials) served as post-stimulation measures of mental rotation performance. In total the experiment had a duration of approximately 80 min (Figure 1A).

### Debriefing

After finishing the experiment, participants filled out a translated version of an adverse effects questionnaire evaluating commonly reported side-effects of transcranial electrical stimulation (TES; Brunoni et al., 2011). Participants had to rate the intensity of adverse effects (1—none, 2—mild, 3—moderate, 4—severe) and how much they were related to the stimulation (1—none, 2—remote, 3—probable, 4—definite). Subsequently, subjects were ask to guess whether they received actual tACS or sham stimulation to ensure they were naive towards their assigned experimental condition. All of them were informed about their experimental condition immediately afterwards.

### Data Analysis

Data analysis was performed using Matlab 2016a (The MathWorks Inc., Natick, MA, USA) and the Fieldtrip toolbox (Oostenveld et al., 2011). For statistical analysis, R 3.2.3 (R Foundation for Statistical Computing, Vienna, Austria) was used.

#### Behavioral Data

Participants’ performance was calculated separately for each block (48 trials, 10 min blocks). Performance during and after stimulation was normalized by performance before tACS to account for inter-individual differences. The resulting percentage values reflect performance increase during each 10 min block relative to baseline. A repeated measurements analysis of variance (rmANOVA) with the within factor block (7 levels, 2 during stimulation, 5 after stimulation) and the between factor condition (2 levels; stimulation vs. sham) was computed. Furthermore, the between factor sex (2 levels) was included to account for possible sex differences.

Reaction times (RTs) were analyzed in a similar manner. To account for the known increase in RTs with larger rotation angles, RTs were first averaged separately for each angle in each block and then normalized with their respective pre-stimulation baseline. Subsequently the normalized RTs were averaged over rotation angles such that the resulting values reflect relative change in RTs over all angles with respect to baseline for each 10 min block. Normalized RTs were finally fed into a rmANOVA with the within factor block (7 levels) and the between subject factors condition (2 levels, *stimulation* vs. *sham*) and sex (2 levels, *males* vs. *females*).

#### EEG

EEG data were resampled to 500 Hz and filtered between 0.3 and 100 Hz. An independent-component-analysis (ICA) was computed on tACS-free EEG signals. ICs reflecting horizontal or vertical eye movements were visually identified and rejected before backprojecting the data into sensor space. EEG data acquired during mental rotation and rest were analyzed separately. Physiological data, acquired during stimulation were not further analyzed due to the large tACS artifact.

To analyze ongoing changes in alpha power each of the pre- and post-stimulation blocks were subdivided into 5 min blocks, such that they consisted of 4 min of mental rotation task and 1 min of resting EEG. This was done to achieve higher temporal resolution of the time-course of ongoing alpha activity. Data during both conditions were analyzed separately. First EEG in each block was segmented into 1 s epochs. Subsequently, a FFT (hanning window, 2 s zero padding) was computed for each segment. Epochs containing residual artifacts were rejected and power-spectra of the first 200 artifact-free segments during mental rotation and the first 43 artifact-free segments during rest were averaged for each of the 5 min blocks. Power in the IAF band (IAF ± 2 Hz) was calculated from the averaged spectra in each block. IAF band power in the post stimulation blocks were normalized by IAF band power during the first 5 min before stimulation (normalization was applied separately for mental rotation and resting data). In accordance with previous approaches (Zaehle et al., 2010; Neuling et al., 2013; Kasten et al., 2016) we focused on electrode Pz for subsequent analysis. In addition mean magnitude squared coherence (Equation 1) in the individual alpha band (IAF ± 2 Hz) between electrode pairs P3-P4 and P7-P8 was calculated for the mental rotation blocks. Coherence in the post-stimulation blocks was normalized by coherence in the pre-stimulation baseline.

(1) $$coh_{xy}(\omega )=\frac{\left|S_{xy}(\omega )\right|}{\sqrt{S_{xx}(\omega )S_{yy}(\omega )}}$$

The magnitude squared coherence for a given frequency (*ω*) is the function of the power spectral densities of two signals *Sxx*(*ω*) and *Syy*(*ω*) and their cross power spectral density (*S_xy_*(*ω*)). The resulting coherence value ranges between 0 (no coherence) and 1 (perfect coherence; Bastos and Schoffelen, 2016). Relative IAF band power and relative coherence were fed into a rmANOVA with within factor *block* (9 levels) and between factors *condition* (2 levels) and *sex* (2 levels). In recent experiments the aftereffect appears to take some minutes until it fully builds up (Neuling et al., 2013; Kasten et al., 2016). Thus, the first block after stimulation was discarded from analysis. To ensure that the effect of tACS is frequency specific and not due to an increase in power in all frequency bands (i.e., caused by changes in impedances), two frequency bands below and above participants individual alpha band were analyzed the same way as described above. For that purpose we choose a lower band from IAF−6 Hz to IAF−3 Hz and an upper band from IAF + 3 to IAF + 6.

To capture event-related changes during the mental rotation task EEG was segmented into 10 s epochs starting 3 s before and ending 7 s after onset of the mental rotation stimulus. Event related alpha synchronization/desynchronization (ERS/ERD) was calculated for each trial. Pfurtscheller and Lopes Da Silva (Pfurtscheller and Lopes da Silva, 1999) defined ERD/ERS as:

(2) $$ERD/ERS=\frac{R-A}{R}\times 100$$

where *A* is the power in the frequency band of interest after stimulus presentation (test period) and *R* is the power during a reference period preceding stimulus presentation (Pfurtscheller and Lopes da Silva, 1999). Positive values indicate ERD during the test period, negative values reflect ERS. Three seconds immediately before and after stimulus onset served as reference and test periods, respectively. Alpha power in both time windows was estimated by computing FFTs on a hanning-tapered sliding window with a fixed length of 1 s moving in steps of 50 ms along each trial. Power in the IAF band (IAF ± 2 Hz) was averaged over the resulting 60 samples for reference and test period. ERD values were computed according to equation 1. ERD values were averaged over trials in each block for each subject and normalized by pre-stimulation baseline. The resulting relative ERD values were fed into a rmANOVA with within factor *block* (5 levels) and between factors *group* (2 levels) and *sex* (2 levels). Please note that for this analysis only blocks after tACS or sham stimulation have been used. Thus, the factor time includes only five levels.

Furthermore, ERPs were calculated for the pre- and post-stimulation periods. To this end, EEG measured at electrodes P7 and P8 was segmented from −0.2 s before to 1 s after the onset of the mental rotation stimulus. Data were baseline corrected by subtracting the mean voltage of the 200 ms interval before stimulus onset from all data points. A low pass-filter at 20 Hz was applied. Artifact-free ERPs were averaged for pre- and post-stimulation periods and over electrodes. For analysis, amplitudes and latencies of three prominent ERP components were extracted from each subject, namely P100, N170 and P300. In contrast to frequency domain analysis post-stimulation data were not normalized by pre-stimulation data, instead pre- and post-stimulation ERPs were compared directly using a rmANOVAs with factors condition (2 levels), sex (2 levels) and block (2 levels).

## Results

### Debriefing

The most frequently reported side-effects (intensity rated 2 or higher) after the experiment were sleepiness (70.6%) and trouble concentrating (64.7%). Although a relatively large proportion of participants associated these adverse effects with the stimulation (47.1% rated sleepiness, 41.2% rated trouble concentrating higher than 2), Wilcoxon rank sum test revealed no differences between groups for any of the ratings (all *p* > 0.1; uncorrected). About 76% of participants indicated that they had been stimulated after finishing the experiment. Fisher’s exact test for count data revealed no significant difference between groups (*OR* = 5.06, *p* = 0.29), suggesting that participants were not aware of their actual experimental condition.

### Mental Rotation Task

#### Performance

To ensure both groups started with similar performance, a Welch two-sample *t*-test was performed to test for differences in baseline performance between *stimulation* and *sham* group. The test revealed no significant differences in baseline performance (*t*_(12.18)_ = −1.4, *p* = 0.18; *M*_stim_ = 84.37%, *SD* = 8.4, *M*_sham_ = 89.35%, *SD* = 5.7).

**Figure 2:**
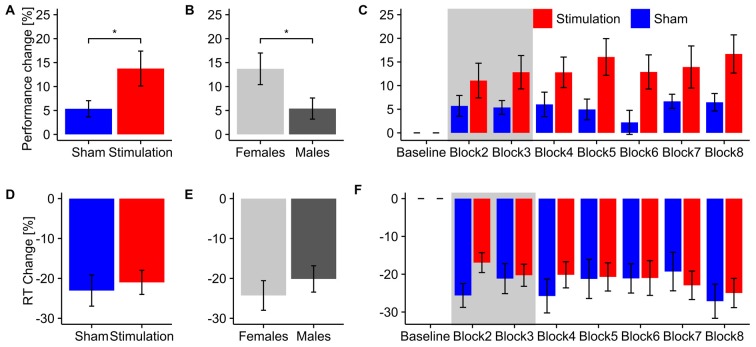
**Behavioral Results.** Top row: overall performance increase of the mental rotation task for **(A)** *stimulation* and *sham* group and **(B)** *male* and *female* subjects. Asterisks depicts significant differences (*<0.05). Error bars depict SEM.** (C)** Time-course of the performance increase for *stimulation* and *sham* group. Gray background indicates blocks during which tACS or sham stimulation was applied. Bottom row: overall change in reaction times for **(D)** *stimulation* and sham group and **(E)** male and female subjects. **(F)** Time-course of reaction time changes for *stimulation* and *sham* group. Gray background indicates blocks during which tACS or sham stimulation was applied. Error bars depict SEM.

Participants in the *stimulation* group exhibited significantly stronger increase in mental rotation performance after stimulation than the *sham* group (*F*_(1,13)_ = 6.04, *p* = 0.029, *η*^2^ = 0.27; Figure 2A). As expected, data revealed an effect of *sex*. *Female* participants showed a stronger performance gain than *males* (*F*_(1,13)_ = 5.88, *p* = 0.031, *η*^2^ = 0.27; Figure 2B). However, there was no interaction of the stimulation with participants’ sex (*condition × sex*: *F*_(1,13)_ = 2.57 *p* = 0.13, *η*^2^ = 0.13). Furthermore, a trend for *block* (*F*_(6,78)_ = 2.46, *p* = 0.07, *η*^2^ = 0.04) has been found. However, please note the relatively small effect size. None of the other interactions reached significance (*condition × block*: *F*_(6,78)_ = 1.55, *p* = 0.21, *η*^2^ = 0.023; *sex × block*: *F*_(6,78)_ = 1.68, *p* = 0.18, *η*^2^ = 0.024; *condition × block × sex*: *F*_(6,78)_ = 0.93, *p* = 0.44, *η*^2^ = 0.013). Overall, both experimental groups enhanced performance in the mental rotation task compared to baseline during and after stimulation (*stimulation*: *t*_7_ = 3.77, *p* = 0.007, *d* = 1.33; *sham*: *t*_8_ = .16, *p* = 0.013, *d* = 1.05).

Contrary to our hypothesis a separate rmANOVA exclusively testing performance during tACS revealed a trend towards increased performance for the *stimulation* group already during stimulation (*F*_(1,13)_ = 3.47, *p* = 0.085, *η*^2^ = 0.19) instead of the predicted reduction in performance. Figure 2C illustrates the time course of mental rotation performance for *stimulation* and *sham* group.

#### Reaction Times

The rmANOVA on normalized reaction times revealed neither an effect of condition (*F*_(1,13)_ = 0.21, *p* = 0.66, *η*^2^ = 0.01), *sex* (*F*_(1,13)_ = 0.72, *p* = 0.41, *η*^2^ = 0.04) or *block* (*F*_(6,78)_ = 1.61, *p* = 0.20, *η*^2^ = 0.03) nor any significant interaction (*condition × sex*: *F*_(1,13)_ = 1.32, *p* = 0.027, *η*^2^ = 0.07; *condition × Block*: *F*_(6,78)_ = 1.64, *p* = 0.18, *η*^2^ = 0.03; *sex × block*: *F*_(6,78)_ = 0.65, *p* = 0.60, *η*^2^ = 0.01; *condition × sex × block*: *F*_(6,78)_ = 0.95, *p* = 0.43, *η*^2^ = 0.02). Overall, both groups significantly reduced their reaction times relative to baseline during and after stimulation (*stimulation*: *t*_7_ = 6.96, *p* < 0.001, *d* = 2.46; *sham*: *t*_8_ = 5.90, *d* = 1.97). For an overview of reaction time results see Figures 2D–F.

### Electrophysiological Results

#### Ongoing EEG

##### Ongoing alpha power during mental rotation

**Figure 3:**
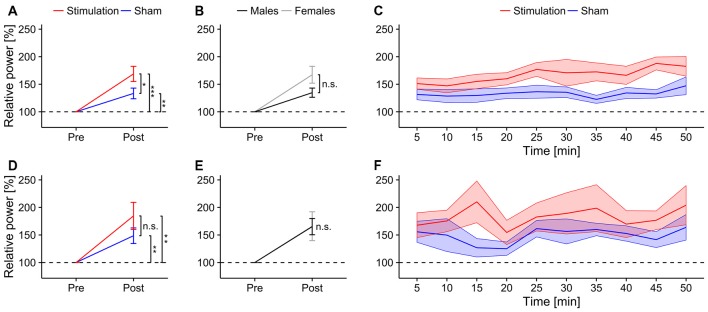
**Ongoing alpha power.** Top row: ongoing alpha power during mental rotation. Error bars and shaded areas depict SEM; asterisks code for significant differences (*<0.05, **<0.01, ***< 0.001). **(A)** Averaged relative alpha power after stimulation for *stimulation* and *sham*. **(B)** Averaged relative alpha power after stimulation for *females* and *males*. **(C)** Time-course of ongoing alpha power after stimulation for *stimulation* and *sham* group. Bottom row: ongoing alpha power during rest. **(D)** Averaged relative alpha power after stimulation for *stimulation* and *sham*. **(E)** Averaged relative alpha power after stimulation for *females* and *males*. **(F)** Time-course of ongoing alpha power after stimulation for *stimulation* and *sham* group.

The rmANOVA revealed a stronger increase in ongoing alpha power during mental rotation in the *stimulation* group compared to *sham* (*F*_(1,13)_ = 4.68, *p* = 0.0496, *η*^2^ = 0.21, Figure 3A). Furthermore, there was a trend towards stronger power increase in the alpha band for *female* subjects compared to *males* (*F*_(1,13)_ = 3.88, *p* = 0.07, *η*^2^ = 0.18, Figure 3B), as well as a significant effect of *block* (*F*_(8,104)_ = 3.28, *p* = 0.002, *η*^2^ = 0.06). None of the interactions were significant (*condition × sex*: *F*_(1,13)_ = 0.07, *p* = 0.79, *η*^2^ < 0.01; *condition × Block*: *F*_(8,104)_ = 1.67, *p* = 0.11, *η*^2^ = 0.02; *sex × block*: *F*_(8,104)_ = 0.57, *p* = 0.80, *η*^2^ = 0.01; *condition × sex × block*: *F*_(8,104)_ = 1.03, *p* = 0.42, *η*^2^ = 0.02). *Post hoc t*-tests against baseline revealed significantly increased alpha power during mental rotation in both groups (*stimulation*: *t*_7_ = 4.98, *p* < 0.001, *d* = 1.76; *sham*: *t*_8_ = 3.45, *p* = 0.004, *d* = 1.15). The time-course of ongoing alpha increase for *stimulation* and *sham* group is depicted in Figure 3C.

Average relative ongoing alpha power during mental rotation was significantly correlated with participants’ increase in performance (*r* = 0.56, *t*_15_ = 2.62, *p* = 0.02; Figure 7A), but not with changes in reaction times (*r* = 0.16, *t*_15_ = 0.63, *p* = 0.53; Figure 7D).

To ensure frequency specific effects of the stimulation, a lower and an upper frequency band around the individual alpha band were analyzed. In the lower band, the rmANOVA only revealed a trend in the interaction between *sex* and *block* (*F*_(8,104)_ = 2.99, *p* = 0.052, *η*^2^ = 0.039). None of the main effects or the other interactions reached significance (*condition*: *F*_(1,13)_ = 1.24, *p* = 0.29, *η*^2^ = 0.07, *sex: F*_(1,13)_ = 0.18, *p* = 0.68, *η*^2^ = 0.01; *block*: *F*_(8,104)_ = 1.74, *p* = 0.18, *η*^2^ = 0.023; *condition × sex: F*_(1,13)_ < 0.01, *p* = 0.92, *η*^2^ < 0.01; *condition × block: F*_(8,104)_ = 0.47, *p* = 0.67, *η*^2^ < 0.01; *condition × sex × block: F*_(8,104)_ = 1.19, *p* = 0.31, *η*^2^ = 0.02). The rmANOVA for the upper band revealed no significant main effects or interactions (*condition: F*_(1,13)_ = 0.49, *p* = 0.50, *η*^2^ = 0.02; *sex: F*_(1,13)_ = 0.01, *p* = 0.92, *η*^2^ < 0.01; *block: F*_(8,104)_ = 1.25, *p* = 0.30, *η*^2^ = 0.04; *condition × sex: F*_(1,13)_ = 0.13, *p* = 0.73, *η*^2^ < 0.01; *condition × block: F*_(8,104)_ = 0.46, *p* = 0.62, *η*^2^ = 0.01; *sex × block: F*_(8,104)_ = 1.60, *p* = 0.22, *η*^2^ = 0.05; *condition × sex × block: F*_(8,104)_ = 0.76, *p* = 0.47, *η*^2^ = 0.02).

##### Ongoing alpha power during rest

The rmANOVA revealed no significant effects of *condition* (*F*_(1,13)_ = 1.63, *p* = 0.22, *η*^2^ = 0.07), *sex* (*F*_(1,13)_ < 0.01, *p* = 0.97, *η*^2^ < 0.01) or *block* (*F*_(8,104)_ = 1.25, *p* = 0.30, *η*^2^ = 0.03) nor any significant interactions (*condition × sex*: *F*_(1,13)_ = 1.43, *p* = 0.25, *η*^2^ = 0.06; *condition × block*: *F*_(8,104)_ = 0.66, *p* = 0.57, *η*^2^ = 0.02; *sex × block*: *F*_(8,104)_ = 0.60, *p* = 0.61, *η*^2^ = 0.02; *condition × sex × block*: *F*_(8,104)_ = 0.40, *p* = 0.75, *η*^2^ = 0.01). However, both groups exhibited significantly increased power during resting periods relative to baseline after stimulation (*stimulation*: *t*_7_ = 3.43, *p* = 0.005, *d* = 1.2; *sham: t*_8_ = 3.43, *p* = 0.004, *d* = 1.14). Overview and time-course of ongoing alpha increase for *stimulation* and *sham* group during rest is depicted in Figures 3D–F. Average relative ongoing alpha power during rest was significantly correlated with participants’ increase in performance (*r* = 0.62, *t*_15_ = 3.05, *p* = 0.008; Figure 7B) but not with changes in reaction times (*r* = −0.21, *t*_15_ = 0.84, *p* = 0.42; Figure 7E).

##### EEG coherence during mental rotation

**Figure 4:**
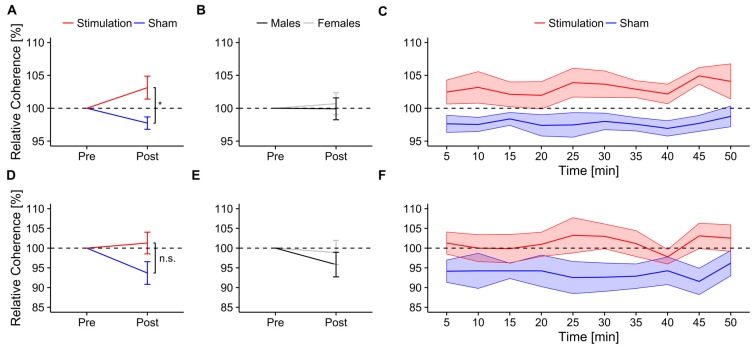
**Ongoing alpha coherence.** Ongoing normalized alpha coherence during mental rotation. Top row: normalized coherence between electrode P3 and P4. Error bars and shaded areas depict SEM; asterisks code for significant differences (*<0.05).** (A)** Averaged normalized coherence after stimulation for *stimulation* and *sham*. **(B)** Averaged normalized coherence after stimulation for *females* and *males*. **(C)** Time-course of normalized coherence after stimulation for *stimulation* and *sham* group. Bottom row: normalized coherence between electrode P7 and P8. **(D)** Averaged normalized coherence after stimulation for *stimulation* and *sham*. **(E)** Averaged normalized coherence after stimulation for *females* and *males*. **(F)** Time-course of normalized coherence after stimulation for *stimulation* and *sham* group.

Relative coherence between electrodes P3 and P4 was significantly higher in the *stimulation* group than in the *sham* group (*F*_(1,13)_ = 7.04, *p* = 0.019, *η*^2^ = 0.28; Figure 4A). There were no significant sex differences (*F*_(1,13)_ = 0.06, *p* = 0.81, *η*^2^ < 0.01; Figure 4B) or effects of *block* (*F*_(1,13)_ = 0.90, *p* = 0.52, *η*^2^ < 0.02) but a significant three-way interaction between *condition, block* and *sex* (*F*_(8,104)_ = 2.20, *p* = 0.033, *η*^2^ = 0.04). None of the other interactions reached significance (*condition × sex*: *F*_(1,13)_ = 0.27, *p* = 0.61, *η*^2^ = 0.02; *condition × block*: *F*_(8,104)_ = 0.54, *p* = 0.83, *η*^2^ = 0.01; *sex × block*: *F*_(8,104)_ = 1.17, *p* = 0.33, *η*^2^ = 0.02). *Post hoc t*-tests show a trend towards increased coherence during mental rotation compared to baseline only for *stimulation* (*t*_7_ = 1.80, *p* = 0.058, *d* = 0.64) but not for *sham* (*t*_8_ = −2.41, *p* = 0.98, *d* = 0.80). Refer to Figure 4C for an overview of the time-course of the coherence change.

The rmANOVA revealed no effects of *condition* (*F*_(1,13)_ = 3.10, *p* = 0.1, *η*^2^ = 0.14; Figure 4D), *sex* (*F*_(1,13)_ = 0.40, *p* = 0.54, *η*^2^ = 0.02; Figure 4E) or *block* (*F*_(8,104)_ = 0.38, *p* = 0.93, *η*^2^ < 0.01) on relative coherence between electrodes P7 and P8. None of the interactions reached significance (*condition × sex*: *F*_(1,13)_ = 0.09, *p* = 0.76, *η*^2^ < 0.01; *condition × block*: *F*_(8,104)_ = 0.87, *p* = 0.54, *η*^2^ = 0.02; *sex × block*: *F*_(8,104)_ = 1.13, *p* = 0.35, *η*^2^ = 0.02; *condition × sex × block*: *F*_(8,104)_ = 0.81, *p* = 0.60, *η*^2^ = 0.02). Neither *stimulation* (*t*_7_ = 0.46, *p* = 0.32, *d* = 0.16), nor *sham* group (*t*_8_ = −2.20, *p* = 0.97, *d* = 0.73) exhibited increased coherence between electrodes P7 and P8 relative to baseline. Refer to Figure 4F for an overview of the time-course of the coherence change.

#### Event-Related EEG

##### Event-related-desynchronization (ERD)

**Figure 5:**
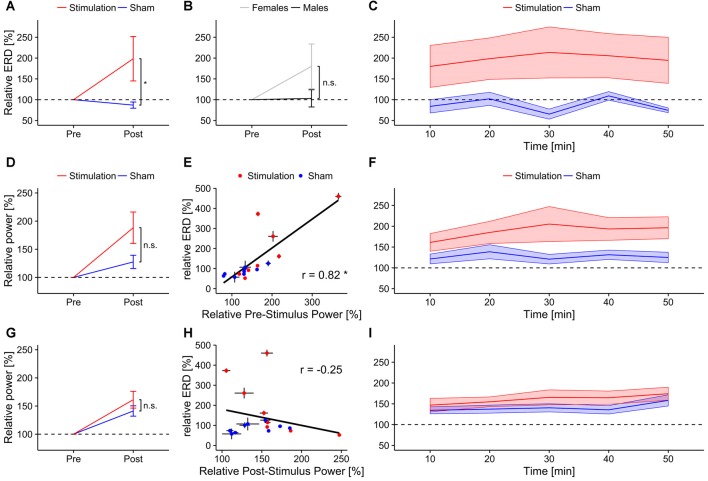
**Event-related-desynchronization (ERD).** Error bars and shaded areas depict SEM; asterisks code for significant differences (*<0.05), n.s. = not significant.** (A)** Overall relative ERD in the individual alpha band for *stimulation* and *sham* group. **(B)** Overall relative ERD in the individual alpha band for *female* and* male* subjects. **(C)** Time-course of relative ERD after stimulation. Bottom rows show relative alpha power 3 s before (reference period; middle row) and after stimulus onset (test period; bottom row). **(D)** Overall relative pre-stimulus alpha power (reference period) for *stimulation* and *sham* group. **(E)** Scatterplot depicting the correlation between relative pre-stimulus alpha power (test period) and relative ERD. **(F)** Time-course of relative pre-stimulus alpha power for *stimulation* and *sham* group. **(G)** Overall relative post-stimulus alpha power for *stimulation* and *sham* group. **(H)** Scatterplot depicting the correlation between relative post-stimulus alpha power and relative ERD. **(I)** Time-course of relative post-stimulus alpha power for *stimulation* and *sham* group.

**Figure 6:**
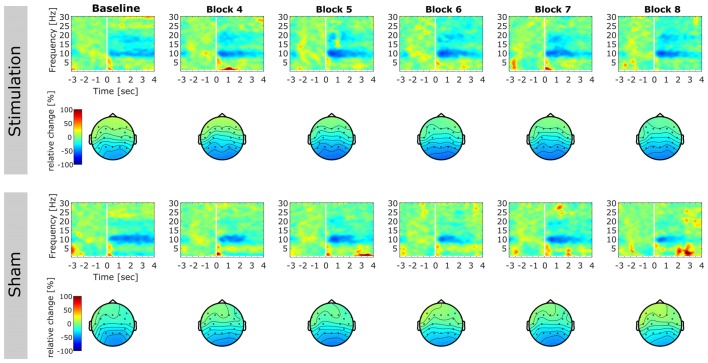
**Event-related relative power change.** Time-frequency representations (TFRs) and topographies reflecting relative change in spectral power after onset of the mental rotation stimulus with respect to baseline (3000 ms prior to stimulus onset until stimulus onset). TFRs are averaged over subjects for each block on electrode Pz. Topographies illustrate relative change in alpha power (8–12 Hz) in the time window 0–3000 ms after stimulus onset. First column displays pre-stimulation baseline. Later columns illustrate post stimulation blocks. Please note that blocks 2 and 3 were performed during stimulation and were discarded from analysis. Top rows: TFRs and topographies of the stimulation group. Bottom rows: TFRs and topographies of the sham group.

ERD increased significantly stronger in the *stimulation* than in the *sham* group (*F*_(1,13)_ = 4.86, *p* = 0.046, *η*^2^ = 0.26; Figure 5A). There were no effects of *sex* (*F*_(1,13)_ = 2.13, *p* = 0.17, *η*^2^ = 0.13; Figure 5B), *block* (*F*_(4,52)_ = 2.05, *p* = 0.13, *η*^2^ =0.01), or significant interactions (*condition × sex*: *F*_(1,13)_ = 1.16, *p* = 0.30, *η*^2^ = 0.08; *condition × block*: *F*_(4,52)_ = 2.15, *p* = 0.12, *η*^2^ = 0.012; *sex × block*: *F*_(4,52)_ = 1.11, *p* = 0.35, *η*^2^ < 0.01; *condition × sex × block*: *F*_(4,52)_ = 0.19, *p* = 0.86, *η*^2^ < 0.01). Only the *stimulation* group exhibited a trend towards increased ERD after stimulation compared to baseline (*stimulation: t*_7_ = 1.84, *p* = 0.053, *d* = 0.65; *sham: t*_8_ = 0.94, *d* = 0.58). The time-course of relative ERD after stimulation is depicted in Figure 5C, time-frequency spectra and ERD topographies are shown in Figure 6.

**Figure 7:**
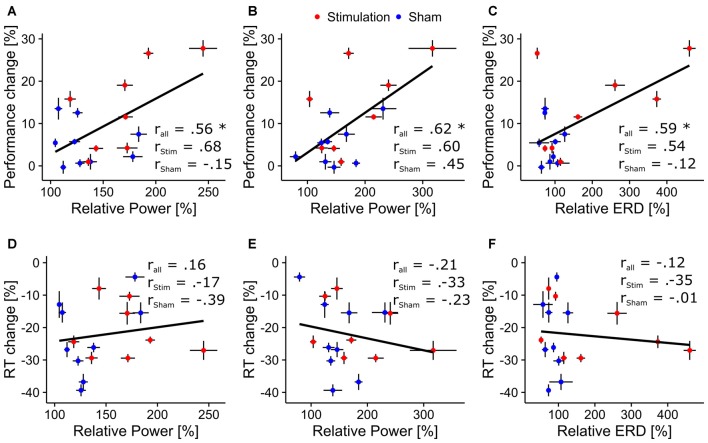
**Correlations between behavioral and physiological measures.** Scatterplots depicting correlations between behavioral and physiological aftereffect measures. Asterisks indicate correlations significantly differing from zero. Black bars around the dots indicate their SEM. Top row: correlation between overall performance increase and **(A)** relative ongoing alpha power during mental rotation, **(B)** relative ongoing alpha power during rest, **(C)** relative ERD. Bottom row: correlation between overall change in reaction times and **(D)** relative alpha power during mental rotation, **(E)** relative alpha power during rest, **(F)** relative ERD.

Relative ERD after stimulation was significantly correlated with participants’ performance increase (*r* = 0.59, *t*_15_ = 2.83, *p* = 0.01; Figure 7C), but not with changes in reaction times (*r* = −0.12, *t*_15_ = 0.45, *p* = 0.65; Figure 7F).

ERD values contain information about the relation of oscillatory activity before (reference period) and after stimulus onset (test period; see Equation 2). Therefore, the observed effect on ERD can be driven by either an increase of alpha power in the reference period, by a decrease of alpha power in the test period or a combination thereof. To further resolve to what extent changes in oscillatory activity in reference and test periods caused the effect the raw spectra used for the calculation of ERD values were extracted and separately analyzed following the previous approach (averaging for each block and normalization with the pre stimulation baseline) and analyzed using rmANOVAs with factors *condition* (2 levels, *stimulation* vs. *sham*) and *time* (5 levels). The factor *sex* was not included as it did not yield significant results in the ERD analysis.

The rmANOVA on alpha power in the reference period revealed a trend for the factor *condition* (*F*_(1,15)_ = 4.37, *p* = 0.054, *η*^2^ = 0.2) but no effect of *block* (*F*_(4,60)_ = 2.35, *p* = 0.11, *η*^2^ = 0.02) and no interaction (*condition × block*: *F*_(4,60)_ = 2.38, *p* = 0.11, *η*^2^ = 0.02, Figures 5D,F). For alpha power in the test period, a significant effect of *block* (*F*_(4,60)_ = 6.66, *p* < 0.001, *η*^2^ = 0.06) but no effect of condition was found (*F*_(1,15)_ = 1.38, *p* = 0.26, *η*^2^ = 0.07). The analysis did not reveal a significant interaction (*F*_(4,60)_ = 0.84, *p* = 0.50, *η*^2^ < 0.01, Figures 5G,I). Only relative reference period alpha power was significantly correlated with the change in ERD (*r* = 0.82, *t*_15_ = 5.47, *p* < 0.001; Figure 5E) but not test period alpha power (*r* = −0.25, *t*_15_ = −0.98, *p* = 0.34; Figure 5H).

##### Event-related potentials (ERP)

**Figure 8:**
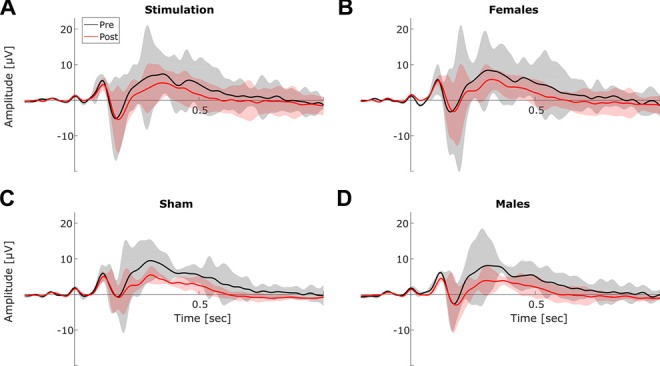
**Event-related-potentials (ERPs).** Grand average ERPs before (black) and after stimulation (red). Shaded areas depict SEM. **(A)** Averaged ERPs before vs. after stimulation for *stimulation* group. **(B)** Averaged ERPs before vs. after stimulation for *female* subjects. **(C)** Averaged ERPs before vs. after stimulation for *sham* group. **(D)** Averaged ERPs before vs. after stimulation for *male* subjects.

Statistical analysis of ERP components revealed significant main effects of *block* for P100 amplitude (*F*_(1,13)_ = 6.18, *p* = 0.03, *η*^2^ = 0.12) and latency (*F*_(1,13)_ = 9.83, *p* = 0.007, *η*^2^ = 0.03), as well as for N170 latency (*F*_(1,13)_ = 5.93, *p* = 0.03, *η*^2^ = 0.03) and for P300 amplitude (*F*_(1,13)_ = 43.72, *p* < 0.001, *η*^2^ = 0.29). Furthermore, analysis revealed a significant effect of *sex* on P100 latency (*F*_(1,13)_ = 8.90, *p* = 0.01, *η*^2^ = 0.40). However there were no significant tACS related changes (no *condition × block* interactions) in any of the extracted ERP components (all *p* > 0.18). The full results of the ERP analysis are summarized in Table 1. Refer to Figure 8 for an overview of pre- and post-stimulation ERPs. Please note, that in contrast to the frequency domain analysis post-stimulation data were not normalized by pre-stimulation data. Instead, pre- and post-stimulation ERPs were compared directly using rmANOVAs with factors *condition* (2 levels), *sex* (2 levels) and *block* (2 levels). Thus, an effect of tACS would show up as an interaction of the factors *condition* and *block*.

**Table 1:** **Analysis of variance (ANOVA) results of event-related-potential (ERP) analysis**.

	*F*	*p*	*η*^2^		*F*	*p*	*η*^2^
**P100 Amplitude**				**P100 Latency**			
*Condition*	0.99	0.34	0.05	*Condition*	0.35	0.56	0.02
*Sex*	2.46	0.14	0.12	*Sex*	8.90	0.01*	0.39
*Block*	6.17	0.03*	0.12	*Block*	9.83	0.007*	0.03
*Condition × Sex*	1.20	0.29	0.06	*Condition × Sex*	0.13	0.72	<0.01
*Condition × Block*	<0.01	0.94	<0.01	*Condition × Block*	0.22	0.65	<0.01
*Sex × Block*	1.36	0.26	0.02	*Sex × Block*	1.45	0.25	<0.01
*Condition × Sex × Block*	0.47	0.50	0.01	*Condition × Sex × Block*	0.20	0.67	<0.01
**N170 Amplitude**				**N170 Latency**			
*Condition*	2.42	0.14	0.15	*Condition*	0.06	0.80	<0.01
*Sex*	0.55	0.47	0.03	*Sex*	1.97	0.18	0.12
*Block*	0.08	0.78	<0.01	*Block*	5.93	0.03*	0.03
*Condition × Sex*	0.08	0.77	<0.01	*Condition × Sex*	2.41	0.14	0.15
*Condition × Block*	0.93	0.35	<0.01	*Condition × Block*	0.40	0.54	<0.01
*Sex × Block*	4.27	0.06^T^	<0.01	*Sex × Block*	<0.01	0.97	<0.01
*Condition × Sex × Block*	0.19	0.67	<0.01	*Condition × Sex × Block*	0.01	0.92	<0.01
**P300 Amplitude**				**P300 Latency**			
*Condition*	0.65	0.43	0.04	*Condition*	0.03	0.87	<0.01
*Sex*	0.48	0.50	0.03	*Sex*	1.50	0.24	0.06
*Block*	43.72	<0.001*	0.28	*Block*	0.05	0.81	<0.01
*Condition × Sex*	0.31	0.59	0.02	*Condition × Sex*	0.31	0.59	0.02
*Condition × Block*	1.99	0.18	0.02	*Condition × Block*	0.26	0.62	<0.01
*Sex × Block*	0.38	0.55	<0.01	*Sex × Block*	0.94	0.35	0.03
*Condition × Sex × Block*	1.77	0.21	0.02	*Condition × Sex × Block*	2.32	0.15	0.07

*Results of repeated measurements analysis of variance (rmANOVA) on amplitudes and latencies of ERP components (P100, N170 and P300). Baseline ERPs were compared to post-stimulation ERPs. No normalization was applied. Asterisks indicate significant effects. Upper case T indicates trends (*p* < 0.1)*.

## Discussion

So far, research on behavioral effects of tACS mainly focused on online effects of the stimulation. While most studies on physiological effects of tACS rely on aftereffects of the stimulation and performed resting-state measurements (Zaehle et al., 2010; Neuling et al., 2013; Vossen et al., 2015; Kasten et al., 2016). The current study combined a complex task, namely mental rotation, with measurements of the outlasting physiological effects of tACS on alpha oscillations, demonstrating both a behavioral and an electrophysiological aftereffect of tACS in parallel.

While there were no effects on participants’ reaction times, performance in the mental rotation task was significantly enhanced in the stimulation group as compared to sham. This is in accordance with previous results suggesting increased performance with enhanced reference alpha power, but no facilitation of reaction times (Klimesch et al., 2003; Hanslmayr et al., 2005; Zoefel et al., 2011). The behavioral effects were accompanied by changes in ongoing and event-related alpha activity. Ongoing individual alpha power and coherence during mental rotation were significantly increased compared to sham. This extends previous findings obtained during simple auditory or visual vigilance tasks (Zaehle et al., 2010; Neuling et al., 2013; Vossen et al., 2015; Kasten et al., 2016). Furthermore, ERD in the individual alpha band was increased in the stimulation group compared to sham. A more detailed analysis revealed some evidence that the observed effect on ERD is probably driven by an increase in pre-stimulus oscillatory power compared to sham while alpha power after stimulus onset in the stimulation group remained similar to the sham group. It is known from previous research, that the effect of tACS is context dependent (Neuling et al., 2013; Ruhnau et al., 2016). However, in these studies permanent changes in context were compared (stimulation and measurement during eyes-closed vs. eyes-open). The current pattern of results suggests that transient changes in context and stimulation effect can occur even on a single trial level which has to be taken into account as these can potentially mask stimulation effects in physiological measurements. Additionally, the finding is in agreement with the theoretical framework of previous NFT and rTMS studies, which aimed to facilitate ERD and mental rotation performance by enhancing alpha power in a reference period (Klimesch et al., 2003; Hanslmayr et al., 2005; Zoefel et al., 2011).

Surprisingly, an effect of tACS was not evident in the resting periods which intermitted the mental rotation task. However, this null finding might be explained by the relatively small amount of data (43 trials per block) that was available for analysis. Furthermore, it is worth noticing that although not significant, the data still point to the same direction as previous findings (Neuling et al., 2013; Vossen et al., 2015; Kasten et al., 2016). Similar to recent results (Neuling et al., 2013; Kasten et al., 2016), the effect of tACS appears to be limited to the stimulated alpha band, as there were no significant effects on neighboring frequency bands. By applying tACS below participants’ individual sensation threshold, we further ruled out, that the observed effects were due to the exposure of skin sensations or the perception of phosphenes. In contrast to the observed changes in performance and in the frequency domain, ERPs were not systematically modulated by tACS. To explain this finding, it should be considered that our stimulation protocol was designed to target ongoing oscillations in the alpha band and was applied independent of stimulus presentation. The induced oscillations contributing to the P1-N1 complex in ERPs, however, are phase locked to the stimulus presentation and might therefore be unaffected by tACS (Gruber et al., 2005; Klimesch et al., 2007a). A more elaborated design, aligning the tACS waveform with the latency of the to-be targeted ERP component might be able to elicit changes in their amplitude. The prominent decrease in P300 amplitude over time is in line with previous research suggesting P300 habituation when a task becomes more automatic and requires less attentional resources (Courchesne, 1978; Romero and Polich, 1996; Ravden and Polich, 1998).

The widely observed sex differences in mental rotation (Linn and Petersen, 1985; Voyer et al., 1995) were also evident in the current data. Female subjects exhibited stronger improvement in the mental rotation task compared to men. Similar observations have been previously made in studies with children and adolescents suggesting that females have more benefits from training and repetition in the domain of mental rotation than males (Neubauer et al., 2010; Tzuriel and Egozi, 2010). However, this is possibly due to lower initial performance and thus more potential for improvement. In addition, females also exhibited a trend towards stronger enhancement of ongoing alpha activity during mental rotation which vanishes during the resting periods, providing a physiological correlate of the aforementioned performance gain. The current results do not suggest that the effects of tACS were modulated by participants’ sex. There is growing evidence that the effects of tACS and brain stimulation in general are highly dependent on the context of application (Silvanto et al., 2008; Feurra et al., 2013; Neuling et al., 2013; Ruhnau et al., 2016). For female subjects, alpha oscillations have been found to be modulated by menstrual cycle (Brötzner et al., 2014) offering a potential source of variance that has hardly been controlled for so far. Furthermore, sex differences and menstrual cycle are among the factors determining the induction of cortical plasticity using other non-invasive brain stimulation techniques such as rTMS or tDCS (Ridding and Ziemann, 2010). Thus, while there was no evidence for an overall interaction between participants’ sex and the tACS effects in the current study, the possibility that tACS effects of females might be modulated by the menstrual cycle cannot completely be ruled out; especially as the current experiment was not tailored to explicitly study sex differences and is therefore possibly underpowered to detect moderate influences of participants’ sex.

Contrary to our initial hypothesis, there was no evidence for a decrease in performance during tACS application. If at all, mental rotation performance of the stimulation group rather increased already during tACS. This is surprising given that decreased ERD was apparent in previous experiments investigating online effects of tACS (Neuling et al., 2015; Vosskuhl et al., 2016). However, it should be acknowledged that this finding was rather a visual observation and not statistically tested or indirectly inferred from a reduction of BOLD signal strength, respectively. Furthermore, both studies utilized a different type of task than the current study (visual change detection task). An important prerequisite for successful entrainment is the presence of a self-sustained oscillator (Pikovsky et al., 2003). Comparing our mental rotation task with the visual change detection task used by Neuling et al. (2015) and Vosskuhl et al. (2016) it is likely that mental rotation involves much stronger ERD up to a complete blocking of alpha oscillations during task execution; thus offering no possibility for entrainment. It is known that ERD in the alpha band is modulated by task demands and complexity, with more demanding tasks resulting in facilitated ERD (Van Winsun et al., 1984; Boiten et al., 1992; Dujardin et al., 1995). During a visual change detection task, relatively simple stimuli (the rotation of the fixation cross) are used and the amount of cognitive load for task execution is low. Thus, residual alpha activity might still be present after stimulus onset offering the possibility to be entrained. In the first case ERD would be increased during tACS due to enhanced pre-stimulus alpha power and subsequent vanishing thereof after stimulus onset. In the latter case ERD would be decreased due to entrained residual alpha activity in the pre- and post-stimulus period. Unfortunately, the current experiment was not optimized for tACS artifact removal techniques applying template subtraction combined with PCA (Helfrich et al., 2014b). We utilized less EEG channels and the stimulation frequency was set to participants’ IAF in order to achieve a maximal stimulation effect rather than being tailored to fit to the sampling frequency of the EEG. This is required for optimal artifact removal. For these reasons, it was not possible to reliably reconstruct EEG signals during stimulation and directly investigate the desynchronization patterns. Further experiments optimized for the still challenging task to remove the tACS artifact or a replication of the current findings using fMRI would be beneficial to resolve these seemingly contradicting results.

The current study extends previous results as it demonstrates tACS to elicit a robust and behaviorally relevant aftereffect in the alpha band during a classic mental rotation task (Shepard and Metzler, 1971; Ganis and Kievit, 2015). While the increase of ongoing alpha power during mental rotation was similar to previous studies on the tACS aftereffect (Neuling et al., 2013; Kasten et al., 2016), the improvement of mental rotation performance was comparable to studies using rTMS or five consecutive days of NFT, respectively (Klimesch et al., 2003; Zoefel et al., 2011). The current study employed a Cz-Oz montage to modulate subjects’ oscillatory activity in the alpha band in posterior brain regions. However, we did not control for other possible montages. Klimesch et al. (2003) found similar effects of rTMS applied over frontal and parietal cortex on mental rotation performance. Thus, it might be possible that the effects reported in the current experiment might also be achieved with other (i.e., frontal-) montages.

In relation to previous tACS studies, the current experiment achieved relatively strong effect sizes with regard to participants performance even though stimulation intensity was on average slightly below the 1 mA that have been reported to be beneficial by Schutter and Wischnewski (2016). However, it should be noted that this value is by no means a threshold necessary to achieve effects but rather provides a rough orientation about the relationship between stimulation intensities and effect sizes that are to be expected. Especially in combination with the benefits of individually guided stimulation at participants’ IAF, results appear to fall in a reasonable range as less energy is needed to entrain an oscillation the closer the driving frequency is to the intrinsic frequency of the oscillator (Pikovsky et al., 2003; Schutter and Wischnewski, 2016). Given the reliable effects of tACS, it might be a suitable method to complement or substitute NFT in experimental as well as in clinical settings. However, direct comparisons of the effects of tACS and NFT are yet missing as well as studies investigating how far paradigms combining NFT with tACS stimulation might lead to stronger or faster effects than each of the methods alone.

We would like to encourage further research to put a stronger emphasis on outlasting behavioral and physiological effects during the investigation of tACS. So far, the vast majority of studies carried out post stimulation measurements of only few minutes, if at all (Veniero et al., 2015). Prolonged paradigms monitoring longer periods of task performance and/or physiological changes after tACS might add additional insights to the mechanisms and long-term effects of tACS. However, when adapting this approach several crucial aspects should be taken into account. While the current study was carried out to target the amplitude of alpha oscillations and its task related modulations (ERD), tACS is potentially capable to modulate several other properties of an oscillation, such as its frequency (Vosskuhl et al., 2015), coherence (Helfrich et al., 2014a; Strüber et al., 2014) or phase (Neuling et al., 2012a). However, depending on the targeted modality aftereffects are more or less likely to occur. Especially when directly modulating the frequency of an oscillation, synchronization theory would predict neural oscillators to return to their intrinsic eigenfrequency shortly after the external driving force (the stimulation) is switched off (Pikovsky et al., 2003). Thus, no sustained changes of frequency are to be expected. Indeed, this pattern has been recently observed in a study tailored to recover EEG signals during tACS by means of template subtraction and PCA (Helfrich et al., 2014b). When targeting amplitude or coherence, aftereffects are produced quite reliably (Zaehle et al., 2010; Neuling et al., 2013; Helfrich et al., 2014a; Strüber et al., 2015; Veniero et al., 2015; Vossen et al., 2015). Complimented by the current findings, we conclude that the tACS aftereffect can potentially be used to study causal relationships between behavior and a variety of properties of brain oscillations such as coherence or amplitude but not their frequency. The current results provide first evidence that a prolonged effect of tACS can be induced during complex tasks. However, it remains unclear to what extent the aftereffect of tACS might interact with task complexity as this factor was not varied in the current design. In a next step, it would be desirable to directly compare different tasks with varying levels of complexity to further understand under which conditions and to which degree of complexity aftereffects can be induced with tACS.

## Author Contributions

FHK acquired and analyzed the data. FHK and CSH designed the study and wrote the article.

## Conflict of Interest Statement

The authors declare that the research was conducted in the absence of any commercial or financial relationships that could be construed as a potential conflict of interest.
